# Blood culture contamination rates at different level healthcare institutions in the Western Cape, South Africa

**DOI:** 10.4102/sajid.v35i1.222

**Published:** 2020-12-11

**Authors:** Christoffel J. Opperman, Banyana Baloyi, Sipho Dlamini, Nazlee Samodien

**Affiliations:** 1 Division of Medical Microbiology, Department of Pathology, Faculty of Health Sciences, National Health Laboratory Service, University of Cape Town, Cape Town, South Africa; 2 Faculty of Health Sciences, University of Cape Town, Cape Town, South Africa; 3 Division of Infectious Diseases and HIV Medicine, Department of Medicine, University of Cape Town, Cape Town, South Africa; 4 Division of Medical Microbiology, National Health Laboratory Service, University of Cape Town and Groote Schuur Hospital, Cape Town, South Africa

**Keywords:** bacterial infections, blood culture, contamination, education, intervention

## Abstract

Sterile blood culture (BC) collection procedures are important to prevent the consequences of contamination, namely, prolonged patient hospitalisation, unnecessary antimicrobial exposure and an increase in hospital costs. Blood culture contamination rates were determined at different hospitals in the Cape Metropole over a 3-year period. Study findings showed that contaminated BCs have a financial impact on the healthcare system and contamination rates remain above accepted international standards, except in the presence of a phlebotomist team. High BC contamination rates might be reduced by the implementation of cost-effective educational intervention programmes, which reminds healthcare workers to collect BC samples aseptically.

## Introduction

Globally blood stream infections result in high morbidity and an overall crude mortality of 15% – 30%.^1^ Blood cultures (BCs) are essential in the diagnosis of bacteraemias and for the guidance of definitive antibiotic therapy. Blood culture contamination is defined as the culturing of organisms that would normally constitute normal skin flora (Coagulase-negative *staphylococci* [CoNS], *Cutibacterium* spp., *Micrococcus* species, *Bacillu*s spp., *Corynebacterium* spp., *Aerococcus* species and *Viridans streptococci*) from a BC sample.^2^ It is well known that non-sterile collection of BCs (contamination) results in additional laboratory workload, unnecessary antibiotic exposure, prolonged hospitalisation, increase in hospital-acquired infections, delayed patient management and increased hospital costs.^3^ The Clinical and Laboratory Standards Institute (CLSI) states that the BC contamination rate should be 3% or less.^4^ An audit at a district hospital in Cape Town, South Africa (SA), showed a high contamination rate of 4.5% annually.^5^ Although the findings may not be representative of the whole country, it indicates a need for cost-effective interventions to reduce BC contamination. To date, there has not been a study of the overall BC contamination rates in SA on a large scale or over an extended time interval.

An intervention such as teaching healthcare professionals how to take blood samples aseptically may reduce the rates of BC contamination and minimise wasteful expenditure.^6^ We evaluate the possible effect of such an intervention at Groote Schuur Hospital (GSH) and calculate the BC contamination rates from primary healthcare facilities to tertiary-level hospitals, including a private facility.

## Methodology

### Setting

The National Health Laboratory Service (NHLS) laboratory is situated within GSH, Cape Town, SA. This laboratory provides services to approximately 80% of the population in the hospital’s drainage area (Southern, Klipfontein, Mitchells Plain and Western health districts), with numerous facilities including 58 primary healthcare facilities, 3 district-, 2 regional- and 2 tertiary-level hospitals and 1 private facility. The private academic hospital is a collaboration between the private healthcare sector, the University of Cape Town and GSH. Through this collaboration, the NHLS at GSH receives and processes a portion of their specimens. All BCs submitted to GSH, NHLS were included in the study and only processed at this laboratory. Both hospitalised patients and those presenting from a community setting were included in the analysis.

### Definitions

A contaminated BC was defined as a sample containing 1 or more of the following ‘skin flora’: CoNS, *Cutibacterium* spp., *Micrococcus* species, *Bacillu*s spp., *Corynebacterium* spp., *Aerococcus* species and *Viridans streptococci* unless there was a possibility of being clinically significant. Electronic laboratory request forms available on the laboratory information system (TrackCare, Version L2016, InterSystems, Sydney, Australia) were reviewed for clinical information to infer clinical significance. Clinical cases of suspected infective endocarditis, in the presence of prosthetic material or devices, an association with indwelling lines and catheters, neutropenic patients or when an organism was recurrent from samples obtained from different venepuncture sites were excluded as contaminant BCs even if these organisms were present. Blood cultures that included a pathogen as well as a contaminating microorganism were considered clinically significant and were not included in the analysis. A polymicrobial BC with more than one contaminating organism present from a solitary venepuncture site was assigned to a single contaminated BC drawn. Blood inoculated into multiple BC bottles from the same draw was analysed as a single event. No standard criteria for collecting BCs exist amongst healthcare institutions. However, the guidelines formulated by Ntusi et al. are recommended.^7^ Paediatric collected samples in this study referred to individuals younger than 18 years.

### Data analysis

Blood culture data between 01 January 2016 and 31 December 2018 were retrospectively extracted from the laboratory information system at the NHLS, based at GSH. The rates of BC contamination in GSH were compared with that of surrounding health facilities on a department and hospital level. Institutional contamination rates were defined as the number of contaminated BCs divided by the total amount of BCs taken. The contaminating microorganisms were identified amongst the total amount of BCs collected during the period investigated.

Shapiro–Wilk and Levene’s test were used to confirm normal distribution of data and homogeneity of variance amongst groups. Analysis of variance (ANOVA) was run to examine the effect of department, age, and hospital level on BC contamination rates. A *p*-value ≤ 0.05 was deemed to be statistically significant. Correction for multiple comparisons was done with Bonferroni post-hoc ANOVA adjustment. Data were imported into Stata 16.1 (StataCorp, College Station, TX, USA) for statistical analysis and stored securely on a computer, password protected and only available to the researchers.

### Cost analysis

Only direct item costs (syringes, needles, sterile cloves, cleaning solution, cleaning packs and blood culture bottle) and laboratory expenses (culture incubation, media plates, biochemical reagents and microscopy) of analysing individual contaminated samples were considered in the cost analysis. This excluded any additional costs related to patient management as a result of a contaminated BC sample. Cost analysis was done on a department and hospital level from accumulative data over a 3-year period.

### Ethical consideration

Ethical approval to conduct the study was obtained from the Research Ethics Committee (HREC: Ref. No. 347/2019) of the University of Cape Town, as well as GSH, Department of Health, Western Cape.

## Results

### Data analysis

**FIGURE 1:**
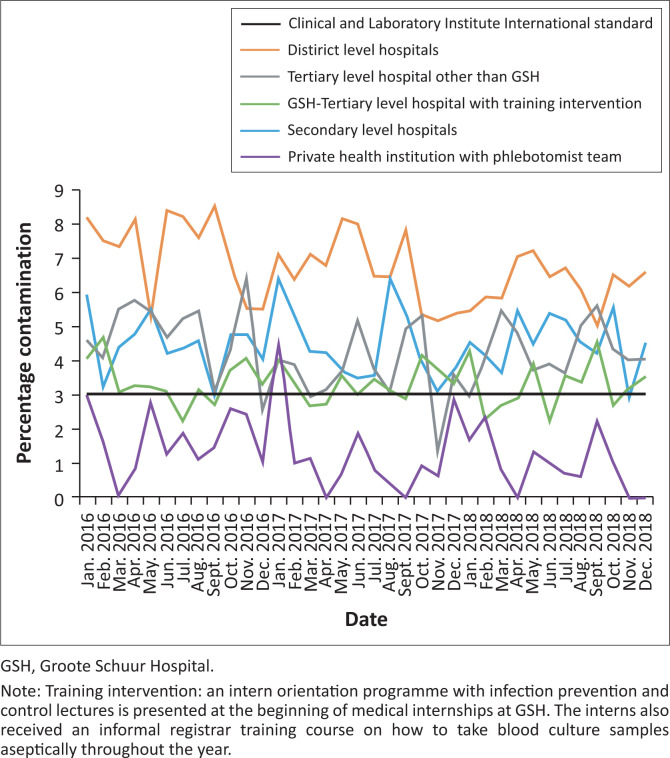
Blood culture contamination rates in different level hospitals with and without the educational intervention compared to the Clinical and Laboratory Institute International standard benchmark of 3%, between 01 January 2016 and 31 December 2018.

Over a 36-month period (01 January 2016–31 December 2018), the BC contamination rate in GSH ranged from 2.2% to 4.5% per month, with an average BC contamination rate of 3.3% over the 3 years. The contamination rate was below 3% during 10 of the 36 months of the study period. In comparison, a private hospital facility with a phlebotomist team had an average contamination rate of 1.3%, a tertiary-level hospital other than GSH 4.3%, secondary-level hospitals 4.5% and district hospitals 6.7%. The district-level hospitals had 26 months with contamination rates twice above the CLSI standards, whilst the phlebotomist team only crossed that barrier once during a 3-year period (Figure 1). In total, the NHLS, GSH microbiology laboratory received 126 490 BC samples over the 3 years. From the BCs submitted the mean age of patients was 32.6 years (standard deviation [SD] 25.1 years), whilst 49.6% of the patients were men.

**FIGURE 2:**
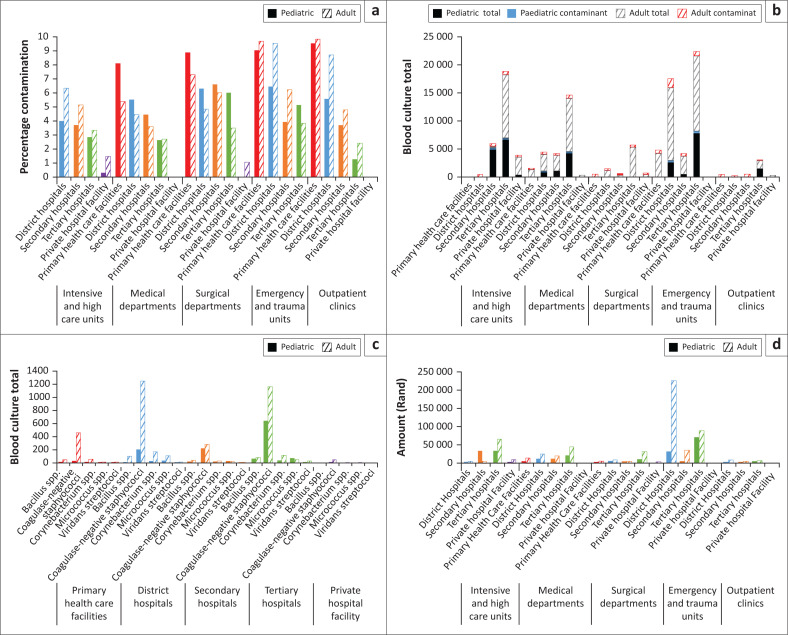
(a) Blood culture contamination rates at different level hospitals and departments in the Cape Town Metropole; (b) Total amount of blood cultures collected with the proportion of contaminating microorganisms cultured; (c) Contaminating microorganisms from processed blood cultures; (d) Blood culture collection consumables and laboratory culturing costs at the National Health Laboratory Service, Groote Schuur Hospital. The study period is between 01 January 2016 and 31 December 2018. The private hospital facility has no emergency or trauma unit. Primary healthcare facilities have no intensive care units.

There was no significant interaction between the effects of department and age (*F*[4,35] = 0.46, *p* = 0.768), age and hospital level (*F*[4,35] = 0.34, *p* = 0.841) or department and hospital level (*F*[14,22] = 1.68, *p* = 0.133) on the BC contamination rate. Two-way ANOVA analysis showed a significant difference in the BC contamination rate and hospital levels (*p* < 0.001) as well as BC contamination rate and departments (*p* < 0.01). Age did not have a significant effect on the BC contamination rate. The model was saturated for a three-way ANOVA interaction analysis. Post-hoc ANOVA (Bonferroni adjusted) analysis illustrated that tertiary hospitals had lower BC contamination rates than secondary (difference in mean = 6.6, *p* < 0.05) and district (difference in mean = 12.2, *p* < 0.001) level hospitals and the primary healthcare facilities (difference in mean = 18.4, *p* < 0.001); the private hospital facility had a lower BC contamination rate than tertiary (difference in mean = 9.0, *p* < 0.01), secondary (difference in mean = 15.6, *p* < 0.001) and district-level hospitals (difference in mean = 21.2, *p* < 0.001) and the primary healthcare facilities (difference in mean = 27.4, *p* < 0.001). The BC contamination rate was the highest amongst the surgical and emergency units, with a mean rate of 10.1% and 13.4%, respectively (Figure 2a), compared to the intensive care unit (ICU) (6.7%), medical (7.3%) and outpatient department (9.4%) over the 3 years. The emergency and trauma unit collected 38.6% (48 798) of all the BCs submitted over the 3-year period, whilst the surgical department only submitted 6.9% (8691). Tertiary hospitals gathered more than half of the study BCs (50.8%, 64 300). Although 73.3% (2833) of all the outpatient and clinic BCs was sent from the two tertiary hospitals, they showed the lowest BC contamination rate amongst all the hospital levels, apart from the private hospital, for this section of the analysis (Figure 2b).

In total, 4279 (77.6%) of the contaminating microorganisms cultured (Figure 2c) over the 3-year period were CoNS (8.9% from primary healthcare facilities, 26.1% from district-level hospitals, 9.2% from secondary-level hospitals, 32.3% from tertiary-level hospitals and 0.9% from the private hospital facility). The hospital ward where a BC was collected was not stated on 10 554 (8.3%) of the laboratory forms received.

### Cost analysis

The cost for processing contaminated BCs over the 3 years amounted to R1 017 576. From this amount R903 965 was allocated to a ward assigned. Adult patient samples accounted for 73.5% (R664 460) of these costs. Tertiary-level hospitals carried the bulk of the expenses at R367 062 (40.6%), whilst R531 165 (58.8%) was allocated towards emergency and trauma units for processing contaminated samples (Figure 2d).

## Discussion

The average BC contamination rate in GSH was 3.3%, which is above the CLSI benchmark of 3%. Some studies have suggested that higher contamination rates are acceptable in teaching facilities.^8^ Various factors may contribute to high BC contamination rates. These include inadequate use of skin antiseptics prior to blood collection, blood collection from a pre-existing line, infrequent sterile technique training with a poor appreciation of the consequences of contamination, uncooperative patients, difficult patient anatomical venepuncture sites and hospitals having a high tolerance rate for BC contamination.^9^

The contamination rates in the private hospital setting with a phlebotomist team were the lowest, with an average rate of 1.3% over the 3 years investigated. The use of a dedicated phlebotomist team leads to a decrease in BC contamination rates as well as a reduction in the associated costs.^8^ Dedicated phlebotomist teams are not feasible in most SA hospitals, burdened with limited staff and financial resources.^10^ District hospitals had the highest rate, ranging from 5.0% to 8.5%. A clinical audit of another district-level hospital in SA showed high BC rates of 4.5% as a result of poor hand hygiene, lack of sterile glove use and inadequate skin antisepsis.^5^

Coagulase-negative staphylococci were the main contaminating organisms found in our study, which is in line with other BC investigations done in SA and around the world.^8,11^ It should be noted that CoNS are an increasingly important pathogen and that there is no true gold standard for differentiating BC contamination from relevant clinical pathogens.^8^

Previous studies have shown that contamination rates are more frequent in the paediatric population.^12^ In the current study age did not affect the BC contamination rate. We observed a substantial lower rate of BC contamination in paediatric wards at tertiary-level hospitals, especially the outpatient departments and clinics. The emergency and trauma units had the highest mean rate of BC contamination over the 3 years (13.4%) and carried almost two-thirds of the expenses (58.85%). The reasons for this are unknown and it needs further investigation. From other studies some of the likely causes include the time pressure of collecting BCs in critically ill patients before resuscitation and obtaining BCs before the first antibiotic dose. Furthermore, high staff and patient turnover and working with uncooperative patients may contribute to increased contamination rates.^13^ Educational interventions, BC collection pacts and changing skin cleaning preparations have been suggested in previous studies to reduce BC contamination in the acute care setting.^14^

An intern orientation programme with infection prevention and control (IPC) lectures has been presented at the beginning of medical internships at GSH for the last 10 years. The interns also received an informal registrar training course on how to take BC samples aseptically throughout the year. Educational programmes have been proven to be effective in decreasing BC contamination rates whilst being cost-effective.^6,15,16^ The BC contamination rates at an ICU in a Northern Ireland hospital were reduced from 9.5% to 3.7% after implementing an educational intervention programme. This study used posters and a 13-min video to increase the staff awareness of BC contamination as well as the proper technique to be used when taking a blood sample.^15^ In another study, one-to-one staff education, observing staff members to ensure that proper techniques were used, resulted in a decrease in the contamination rate from 5.7% to 1.9%.^6^ The educational interventions at GSH may have contributed to the low BC contamination rates observed. This however cannot be confirmed as the BC contamination rates before and after this educational intervention have not been studied. Blood culture educational interventions should have metrics in place to measure the impact of teaching activities. Multiple teaching interactions will most likely be warranted to reinforce core principles, such as taking BCs aseptically instead of single encounters.

Blood culture contamination has a significant impact on pharmacy and laboratory costs.^4,6^ The economic impact of processing these cultures cannot be understated. Over the 3-year period, more than 1 million rand was spent on consumables to collect blood cultures and process them in the laboratory alone. This does not include the costs associated with unnecessary antibiotic therapy, increased length of hospitalisation, treatment of hospital-acquired infections or the time spent by staff members in taking care of patients during the additional hospital stay. Therefore, this value grossly underestimates the true financial impact of contaminated BCs on the healthcare system.

This study has limitations mainly because of the retrospective study design, as the analysis relied on the integrity of the data extracted. No data are available for comparison before the educational interventions that were implemented at GSH. Although educational interventions have shown in the literature to reduce BC contamination rates and save costs, no multivariate analysis was performed to exclude confounding factors in this study, which may have contributed to the low rates of BC contamination at GSH. Future research on this topic should be dedicated to this aspect. The current data look at a department level and not at ward levels; therefore, interventions directed towards meeting discipline-specific challenges could not be developed. In addition, we did not investigate the cause(s) of BC contamination in each department or hospital. Finally, cost analysis or auditing was not done beyond the consumables and laboratory processing costs.

## Conclusion

Overall, BC contamination rates continue to be above the accepted international range amongst healthcare institutions in the Western Cape province, SA. Staff education is central to the reduction of BC contamination, especially in settings without a dedicated phlebotomist team. The low BC contamination rates at GSH (tertiary institution) may be related to the educational IPC interventions and possibly the collection of BCs under the guidance of senior staff. Teaching IPC and sterile BC collection should include variable creative methods (especially in low-resource settings), such as ward posters, videos and one-on-one staff interactions, with metrics in place to measure their impacts. Amongst all the units, the emergency and trauma sections were found to have the highest BC contamination rates, with a large number of BC samples collected. This justifies the need for low-cost, multifaceted interventions in this section of the hospital to reduce contamination rates and lessen the possible additional costs on the healthcare system.
